# The G-force in the genome: Unknowns on the functional flairs of DNA G-quadruplexes

**DOI:** 10.1371/journal.pbio.3003874

**Published:** 2026-07-16

**Authors:** Irene Gallina, Sara N. Richter

**Affiliations:** 1 Department of Molecular Medicine, University of Padova, Padova, Italy; 2 Microbiology and Virology Unit, Padua University Hospital, Padua, Italy

## Abstract

Alternative DNA secondary structures known as G-quadruplexes (G4s) form across all kingdoms of life, regulating transcription, telomere stability, and genome maintenance. Although many putative G4-forming sequences are predicted to exist, it remains unclear which ones fold in cells at any given time and what mechanisms drive their formation. Moreover, while G4s have been primarily linked to transcriptional regulation, how this function is achieved remains to be investigated, and the roles of G4s at other genomic regions remain unclear. This Unsolved Mystery discusses key gaps in our understanding of G4 formation and function and highlights recent mechanistic and methodological advances that are beginning to address them.

## Abstract 2

DNA G-quadruplexes are dynamic four-stranded DNA knots that may control genes, protect genomes, and drive disease, yet scientists still do not fully understand when they form, how they work, and why cells need them. This Unsolved Mystery investigates these questions.

## Introduction

**Fig 1:**
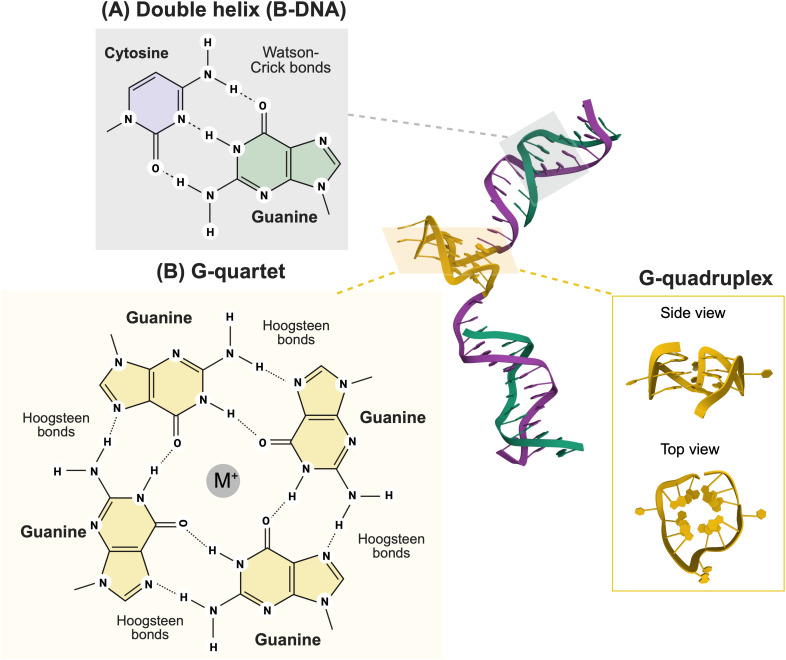
Structure and chemistry of G-quadruplexes (G4s). Structure of a duplex-G4-duplex DNA molecule (image from the RCSB PDB (http://www.rcsb.org/) of PDB ID 8DUT [4]). Duplex DNA is depicted in purple and dark green; G4 DNA is depicted in yellow, and both side and top views of the G4 structure are shown. The displaced strand opposite the G4 has been omitted for clarity. **A**. Chemistry of Watson-Crick hydrogen bonds between complementary cytosine and guanine on duplex B-DNA. **B**. Chemistry of planar Hoogsteen hydrogen bonds between four guanines within a G-tetrad. Guanines in a tetrad are usually coordinated by a monovalent cation such as potassium (K^+^). Created in BioRender. Gallina, I. (2026) https://BioRender.com/mbd2gp3.

The ability of DNA to adopt conformations distinct from the canonical double-helical B-form described by Watson and Crick has been recognized since the early 1980s. In 1988, tracts of consecutive guanines were observed to fold into higher-order structures in the presence of monovalent cations, such as sodium and potassium, in vitro [1]. These structures, later termed G-quadruplexes (G4s), represent one of the non-canonical conformations that nucleic acids can adopt in guanine-rich regions of the genome or in guanine-rich RNA molecules. Specifically, sequences containing four tracks of two or more consecutive guanines can self-assemble into planar G-tetrads through Hoogsteen hydrogen-bond base pairing, and G-tetrads can stack on top of each other to form G4s that, unlike the double helix, appear as four-stranded structures (Fig 1). Following their discovery, the biophysical properties of G4s have been extensively characterized. Structural studies revealed alternative G4 topologies, and in vitro analyses established some correlation between G4 sequence composition, topology, and thermodynamic stability [2,3]. Together, these studies portrayed G4s as highly dynamic structures that, despite their conserved core architecture, can adopt multiple sequence-dependent conformations, suggesting the potential for diverse functions in vivo.

For many years, however, skepticism persisted regarding the physiological relevance of G4s, and their functional significance began to gain broad acceptance only after major advances in computational and experimental approaches. Genome-wide analyses identified a non-random distribution of putative G4-forming sequences across a wide range of organisms [5], implying a functional relevance for G4s. Moreover, the development of structure-specific antibodies enabled the visualization of G4s in living cells and the identification of their location within the genome [6]. Concomitantly, G4 ligands developed in vitro and selectively binding and stabilizing G4s have been exploited in cells to assess the effects of the modulation of G4 folding [7]. These studies collectively confirmed that G4s form in cells and are highly dynamic, with their formation and resolution tightly regulated at different genomic locations at different times of the cell cycle. G4 stability in cells is influenced by multiple factors, including local sequence context, chromatin environment, and transcriptional activity, rendering these structures responsive regulatory elements [8]. Consistently, G4s are predominantly found in functionally important genomic regions, including gene promoters, enhancers, and telomeres [9]. Promoter-G4s are frequently found upstream of transcription start sites (TSSs) and are overrepresented in genes involved in cell cycle regulation, development, and stress responses [9]. At telomeres, G4s have a well-established role in controlling access of telomerase to chromosome ends and in influencing telomere length homeostasis [10].

The biological relevance of G4s is further underscored by the identification of different classes of proteins that recognize, stabilize, or resolve them, including specialized helicases (such as members of the RecQ and DEAH-box families) [11] and G4-binding proteins [12] that modulate G4 stability and enable their reversible formation. Dysregulation of G4 formation or resolution, either through G4-stabilizing ligands or by depletion of G4-resolving helicases, has been associated with replication stress, genomic instability, and aberrant gene expression [13,14], which are all hallmarks of cancer. In this context, G4 stabilization by G4 ligands has emerged as a promising approach for cancer treatment [15]. In fact, ligand-stabilized G4s behave as physical obstacles to DNA replication, inducing DNA breaks, and exhibit synthetic lethality in repair-deficient cancer cells [16]. In addition, ligands targeting telomeric G4s can induce telomere shortening and, consequently, cancer cell death [17]. The importance of G4 regulation in human health is also highlighted by the involvement of enhanced stability of G4s in neurodegenerative diseases and the recent identification of G4 unfolding agents as potential therapeutics [18,19]. Viral genomes also frequently exploit G4 formation to regulate their replication and transcription, making G4s attractive targets for antiviral strategies as well.

Despite nearly four decades of research and major advances in identifying the structural determinants and activity of G4s in cells, fundamental aspects of the biological role of DNA G4s still remain unknown. Although hundreds of thousands of sequences in the human genome possess the intrinsic potential to form G4 structures, only approximately 1% are detected in cells at any given time [6]. This discrepancy may be only partially dependent on the functional regulation of G4 formation in cells, and could also reflect non-functional or highly context-specific G4s. G4s with lower thermostability and low sequence conservation, such as those predicted in the non-transcribed strands of exons, are in fact de-enriched from functional genomic elements such as promoters [20], indicating that they probably do not fold in cells or that their folding is functionally neutral. Approaches that aim to genetically manipulate G4 sequences to observe functional outcomes and G4 mapping approaches in cells are being used to address central questions in the field, including: what determines whether a G4-forming sequence actually folds into a G4 in cells; how are specific sequence features, chromatin context, transcriptional dynamics, and the activity of G4-binding proteins and helicases integrated to regulate G4 formation and persistence; what are the molecular mechanisms by which G4s regulate biological processes, such as transcription? In this Unsolved Mystery, we dissect these and other unresolved issues and discuss emerging strategies aimed at providing definitive answers.

## What determines G4 formation in cells?

**Fig 2:**
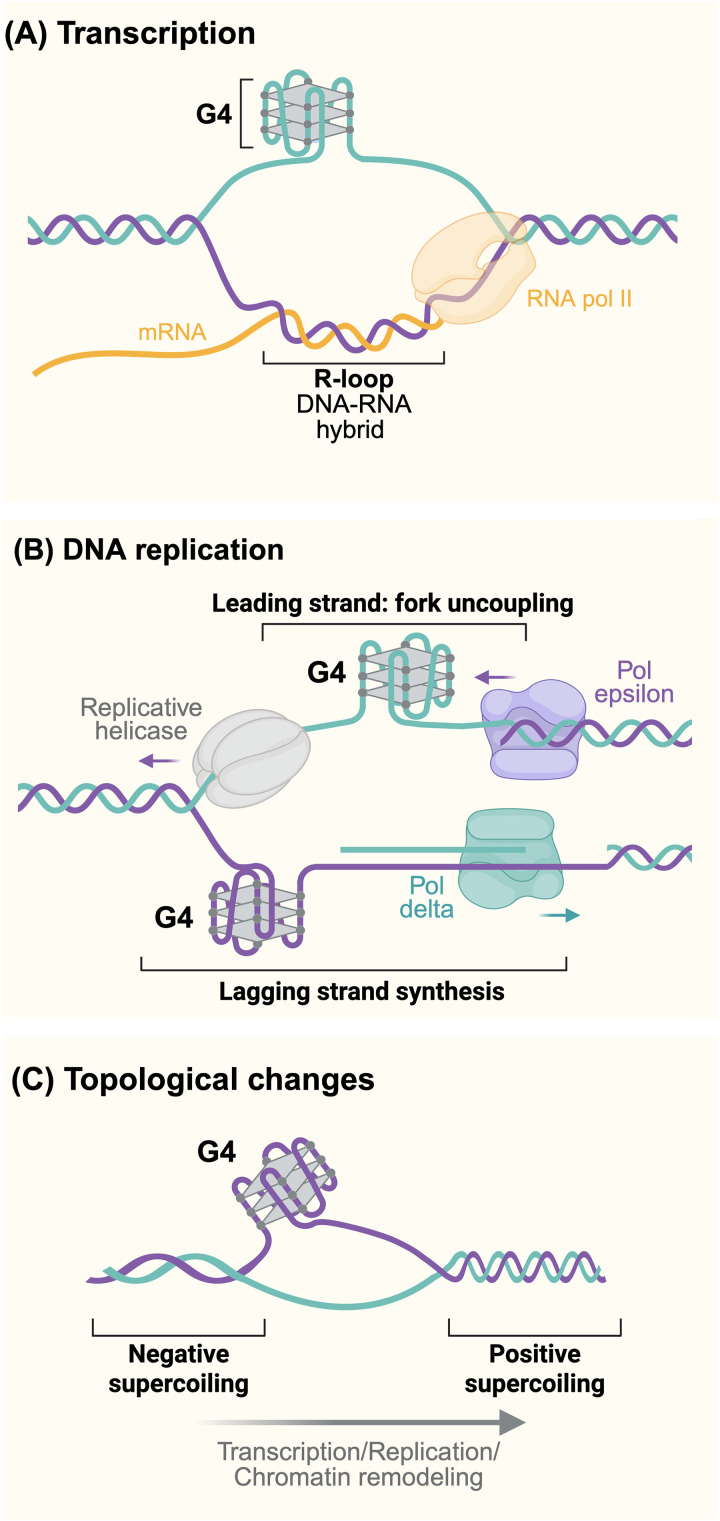
How do G-quadruplexes (G4s) form in cells? A. During transcription, formation of single-stranded DNA at the transcription bubble generated by RNA polymerase II (RNA pol II) facilitates G4 folding, particularly at the transcription start site. G4s are formed at the guanine-rich strand, while the complementary cytosine-rich strand can engage with the nascent mRNA to form an R-loop. R-loops and G4s at the same site stabilize each other, preventing re-annealing of the two melted strands. **B.** During DNA replication, the replicative helicase unwinds the double-stranded DNA and DNA replication proceeds bidirectionally with the leading strand synthesized continuously by DNA polymerase epsilon (pol epsilon) and the lagging strand synthesized discontinuously by a mechanism also involving polymerase delta (pol delta). DNA replication generates a bubble that may favor G4 formation by exposing single-stranded DNA. Single-stranded DNA is formed either on the parental lagging strand or when uncoupling between helicase activity and leading-strand synthesis exposes single-stranded DNA on the leading strand. **C.** Changes in DNA topology. DNA unwinding by both DNA and RNA polymerases and action of chromatin remodelers cause local changes in DNA topology. DNA becomes negatively supercoiled (under-twisted) behind and positively supercoiled (over-twisted) in front of the processing enzyme. Negative supercoiling can cause local melting of the double helix, exposing single-stranded DNA and favoring G4 folding. Created in BioRender. Gallina, I. (2026) https://BioRender.com/7ucn6z2.

While G4 folding in vitro is a thermodynamically passive process driven by DNA sequence and ionic strength, what forces lead to G4 formation in living cells? Inside the nucleus, DNA is predominantly double-stranded and extensively bound by proteins, creating a much more complex and diverse environment than test-tube conditions. It is widely accepted that G4 folding, which involves guanines from the same DNA strand pairing into G-tetrads, requires access to single-stranded DNA. In cells, stretches of single-stranded DNA mainly form during transcription and DNA replication, two processes that transiently unwind the DNA double helix. During transcription initiation, RNA polymerases locally unwind the DNA to create a transcription bubble, in which the DNA is temporarily single-stranded (Fig 2A). Similarly, DNA replication generates stretches of single-stranded DNA, either on the parental lagging strand during unperturbed replication or on the parental leading strand when DNA unwinding by the replicative helicase uncouples from nascent strand synthesis due to obstacles on the template DNA (Fig 2B). Consistent with this model, G4s are predominantly detected at promoters of actively transcribed genes, and recent evidence shows that their presence at sites of active replication increases under conditions of mild replication stress, when single-stranded DNA is generated at the replication fork [21]. In addition to active strand separation, double-stranded DNA can also be transiently melted by local changes in DNA supercoiling, which is the degree to which the DNA helix is twisted around its axis. Negative supercoiling, or underwinding of DNA, which is mainly generated upstream of progressing RNA or DNA polymerases, can destabilize hydrogen bonding between the two DNA strands, generating single-stranded DNA regions [22]. Although negative supercoiling is normally relieved by topoisomerases through DNA cleavage and formation of protein–DNA cleavage complexes [23], it can accumulate locally, for example, at highly transcribed genes [24] or following chromatin remodeling, thereby promoting G4 formation (Fig 2C).

One main problem with this view is that transient generation of single-stranded DNA may not be sufficient to allow G4 folding. Because double-stranded DNA is thermodynamically favored, G4 formation requires conditions in which the single-stranded DNA is prevented from re-annealing with its complementary strand. While the melted strands are physically separated at the replisome during DNA replication [25], this is not the case near TSSs, where most G4s are mapped in cells. So, how are replication-independent G4s stabilized? One possibility is that the strand complementary to the G4-forming sequence is sequestered by folding into an i-motif, a DNA secondary structure formed at cytosine-rich sequences. Although the folding of i-motifs in cells has long been debated, recent genome-wide studies have mapped these structures and shown their enrichment at gene promoters [26]. However, it remains to be established whether i-motifs can co-exist with G4s at the same sites on complementary strands in cells and how this might influence G4 stability and function. A second mechanism involves nascent RNA transcripts, which can hybridize with their DNA template strand generating DNA–RNA hybrids and, by displacing the non-template strand, form R-loops [27]. R-loops preferentially form at G-rich sequences [28] and have been shown to favor stability of G4s folded on the displaced non-template strand. Accordingly, G4s and R-loops are often mapped to the same gene promoter regions [26,27,29], suggesting a coordinated mechanism that might regulate transcription at these sites (see below). Thirdly, G4-binding proteins, including transcription factors such as Nucleolin and SP1, can promote G4 folding by stabilizing specific G-rich conformations and acting as molecular chaperones, lowering the energetic barrier required for G4 formation and shifting the equilibrium from partially folded DNA toward a stable G4 topology [30]. In addition, protein binding can shield the structure from unwinding helicases or prevent re-annealing of the DNA helix, thereby favoring G4 folding and stability. Although no proteins that directly generate G4s from double-stranded DNA have yet been identified, this remains an intriguing possibility, which could also explain G4 formation at closed chromatin sites, which are less common but still reported [31].

Therefore, while multiple players influencing G4 formation and stability have been identified, further studies are needed to define the key drivers regulating the highly dynamic equilibrium between G4 folding and unfolding in cells. Given the complexity of the cellular system, some answers might come from in vitro single-molecule approaches, such as those based on optical or magnetic tweezers [32]. These techniques enable re-constitution of G4 folding on defined DNA substrates and real-time monitoring of folding kinetics upon the addition of RNA molecules or DNA binding proteins, including histones. Moreover, they also allow precise control over nucleic acid composition, protein abundance, and torsional forces, enabling the concomitant investigation of multiple factors influencing G4 folding [33]. A key future challenge will be the refinement of these systems to recapitulate the interactions between the different forces driving G4 folding in a more physiologically complex context.

## How do G4s regulate transcription?

**Fig 3:**
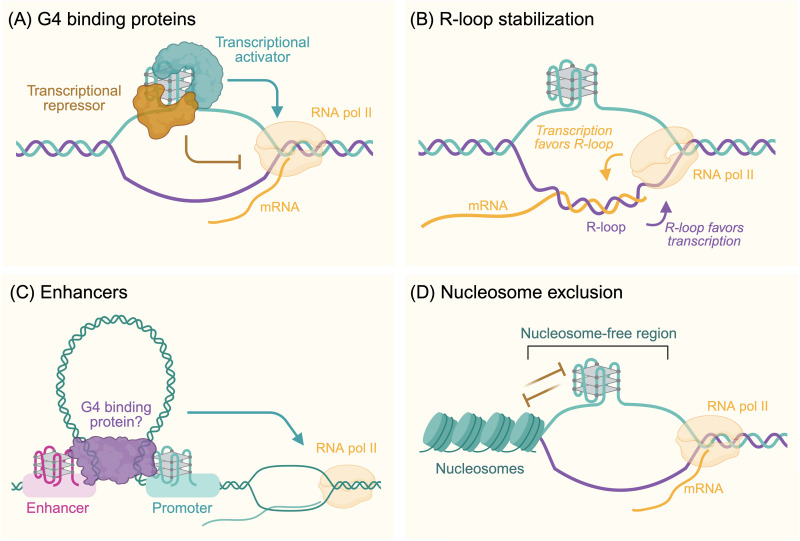
How do G-quadruplexes (G4s) regulate gene expression? A. G4-binding proteins. G4s are hubs for the binding of transcription factors at promoters. Mainly transcriptional activators, but also repressors bind to G4s and modulate transcription by regulating RNA polymerase II (RNA pol II) binding at promoters. **B.** R-loop stabilization. At the transcription start site (TSS), G4s and R-loops reciprocally stabilize each other, sustaining gene expression. R-loop formation, stabilized by G4s on the opposite DNA strand, enhances transcription by RNA pol II (purple arrow). Concomitantly, enhanced transcription and mRNA formation promote R-loop stabilization (yellow arrow). **C.** Enhancers. G4s are enriched at enhancers, intergenic regions that regulate expression of multiple genes. G4s at enhancers regulate DNA looping and promote transcription of multiple gene targets. This function of G4s may be mediated by G4-binding proteins bridging the interaction between G4s at enhancers and G4s at promoters. **D.** Nucleosome exclusion. G4s and nucleosomes are often mutually exclusive. Nucleosomes hinder G4 folding, while folded G4s prevent DNA wrapping around nucleosomes. This may generate nucleosome-free regions at TSSs that promote gene expression by stabilizing G4 folding. Created in BioRender. Gallina, I. (2026) https://BioRender.com/ueg1m5q.

Soon after their discovery in the promoters of oncogenes such as *MYC* and given their genome-wide enrichment and structural conservation at the TSS [34], G4s were implicated in transcriptional regulation, a view now supported by substantial experimental evidence based on quantitative occupancy [35]. In eukaryotes, G4s located at the TSS predominantly promote transcription, although specific G4 conformations were recently shown to induce transcriptional repression [36]. However, the precise mechanisms by which G4s regulate transcription remain incompletely understood, with several non-mutually exclusive models emerging. The first described model considers G4s as binding sites for transcription factors. Extensive evidence has shown binding of transcription factors to G4-forming sequences in vitro and these transcription factors have been mapped at promoters in cells, although a comprehensive analysis of their genome-wide distribution with respect to G4s is missing. Importantly, transcriptional repressors have also been shown to bind to G4s at promoters [37], painting a picture in which G4s act as general hubs for the recruitment of transcriptional regulators [12] (see Fig 3A for a model of G4s as protein-binding hubs). Most G4 binders show high affinity for DNA and a variable degree of selectivity for G4s over single- or double-stranded DNA in vitro; however, it remains to be clarified if the transcription factor binding specificity is more sequence- or G4-dependent in physiological contexts. Notably, these studies are inherently challenging, as mutations needed to abolish G4 folding and determine protein binding specificity to G4s inevitably modify the primary DNA sequence, often affecting transcription factor binding sites, and making it difficult to unambiguously attribute transcriptional effects to G4 folding per se. Other open questions remain regarding how selectivity between G4 binding by transcriptional activators and repressors would be achieved and the extent to which chromatin accessibility affects these processes (see below).

More recently, G4s have also been proposed to act as transcriptional amplifiers, rather than simple transcriptional activators. In this model, G4s are not pre-formed structures that only favor transcriptional activation at promoters. Instead, they fold as a consequence of transcription, triggered by transcription bubble formation, and supported by transcription-dependent negative supercoiling. Once formed, G4s function by sustaining a stable level of gene expression through a positive feedback loop involving engagement of nascent mRNA transcripts in R-loop formation (see Fig 3B for a model of G4s as transcriptional amplifiers). In fact, R-loop stabilization has been shown to increase promoter accessibility to RNA polymerase II and to locally boost transcription [38,39]. This model, which may apply to a subset of G4s, is consistent with the observed enrichment of G4s at highly transcribed genes [9] and with the frequent co-localization of G4s and R-loops [28,37], although direct evidence of whether this co-localization leads to enhanced or stabilized gene expression is still lacking. Importantly, G4s are not only enriched at promoters but are also frequently found at enhancers, intergenic regions that regulate chromatin looping and enhance the expression of multiple gene targets. At these sites, G4s have been proposed to promote long-range transcription by stabilizing chromatin loops, potentially through the formation of long-range intermolecular G4s [40] or by interactions between intermolecular G4s and G4-binding protein complexes that help maintain loop integrity [41] (Fig 3C). However, additional evidence is needed to elucidate the underlying mechanism and to answer key questions in the field, including how G4 regulate enhancer-promoter communication and to what degree G4–enhancer interactions are cell-type specific. While these findings outline plausible mechanisms by which G4s might regulate transcription, a major unresolved question concerns how this activity is modulated and particularly how it is switched off. Is G4 unfolding a consequence of transcriptional downregulation and R-loop dissolution? Or is G4 dissolution at promoters regulated by G4-resolving proteins such as helicases? And if so, how are these helicases themselves controlled to ensure that G4s are unfolded only when transcription needs to be downregulated?

At least some answers may come from one possible model that describes G4s as epigenetic marks, suggesting that these structures can modulate gene expression at a higher regulatory level by influencing chromatin accessibility at promoters, similar to histone modifications and DNA methylation (see Fig 3D for a model of G4s interplay with chromatin state). Promoters are in fact well known to carry euchromatic histone marks that confer an open chromatin conformation, and genome-wide mapping studies reveal an extensive overlap between G4s and euchromatin regions [9]. More specifically, G4s are enriched in nucleosome-depleted regions within promoters [42], consistent with a model in which nucleosomes and G4s are mutually exclusive. Still, a key question remains regarding the causal relationship between G4 folding and open chromatin establishment. Does euchromatin promote G4 formation, and how (see also above), or can G4s actively drive the establishment of euchromatin at promoters? Similarly, does nucleosome positioning determine which promoters are permissive for G4 folding, or does G4 formation itself exclude nucleosomes and thereby render promoters transcriptionally competent? Notably, promoter-associated G4 folding has been recently observed to precede transcriptional activation in cancer cell lines and to be negatively influenced by closed chromatin, supporting the model where G4 formation is a consequence of open chromatin availability [43]. Concomitantly, G4-stabilizing ligands have been observed to induce specific changes in chromatin accessibility [44], in agreement with G4s functioning as epigenetic regulators. Intriguingly, chromatin remodelers that promote open chromatin formation by actively extracting nucleosomes such as SMARCA4 can selectively bind to G4s at promoters [45], implying a possible causal link between G4 folding and the formation of accessible chromatin.

In parallel, guanine and cytosine residues have also been strongly implicated in epigenetic regulation by a different mechanism, which involves chemical modification of the DNA bases by a methyl group. This modification is actively added to cytosine residues in particular by methyltransferases such as DNMT1, with DNA methylation acting as a repressive epigenetic mark that inversely correlates with euchromatin formation and selectively silences gene expression, especially during development [46]. Accordingly, active gene promoters are generally hypomethylated and G4-prone, whereas silenced genomic loci show hypermethylation and a paucity of G4s. Intriguingly, growing evidence points to direct interplay between G4 formation and DNA methylation. On the one hand, G4s appear to negatively impact DNA methylation by locally hindering DNMT1 activity on cytosines at the same genomic locations [47]. On the other hand, cytosine methylation has been shown to locally impair G4 formation, possibly by modulating the stability of the secondary DNA structure [48,49]. Further studies will be required to disentangle these reciprocal interactions, to establish causality and, in the end, to validate the role of G4s as primary determinants of chromatin architecture. This will also be crucial to clarify how G4s are established and maintained during cell differentiation and development, processes in which precise regulation of gene expression is critical for cell identity. In this context, it will be important to determine whether dysregulated G4-dependent mechanisms represent an as-yet-unexplored contributor to developmental disorders.

## Can G4s regulate DNA replication?

As discussed above, G4s can form naturally during bulk DNA replication without an obvious function, and may even hinder replication fork progression (see below). However, it remains to be explored whether G4s have regulatory roles in DNA replication. Although G4s are most frequently mapped at promoters of actively transcribed genes, they are also enriched in other regions of the human genome, namely telomeres and replication origins. Notably, telomeric repeats (TTAGGG in humans), located at the end of eukaryotic chromosomes, were the first DNA sequences shown to fold into G4s [1,50]. Telomeric G4s fold both in the single-stranded 3′ overhang at chromosome ends [51] and within the double-stranded telomeric DNA, where they form on the strand opposite specific R-loops generated by the telomere-specific RNA TERRA [52]. G4s are thought to contribute to telomere homeostasis primarily by regulating telomere length. Proposed mechanisms include facilitating the recruitment of telomere replication factors [53], regulating alternative lengthening of telomeres [54], and controlling telomere end capping [55]. Future studies should aim at defining the spatiotemporal dynamics of G4 formation at telomeres and determining how these structures are modulated throughout the cell cycle. In addition, improved tools to selectively detect and perturb telomeric G4s in cells will be necessary to distinguish the causal roles of G4s in the regulation of telomere homeostasis.

A second, more debated role for G4s in DNA replication concerns their involvement in defining replication origins in humans and other metazoans. Unlike in organisms such as yeast, replication origins in metazoans are not defined by a strict DNA sequence consensus [56]. In this context, putative G4-forming sequences have been identified upstream of a subset of metazoan replication origins [57]. Origins containing these sequences share common features: they are replicated early in S phase and strongly overlap with regions of active transcription. Moreover, G4-forming elements are sufficient to define replication origins when inserted ectopically into the genome, and mutation of these sequences at endogenous origins significantly reduces replication initiation efficiency [58]. Mechanistically, G4s at replication origins have been proposed to define nucleosome-depleted regions necessary for the binding of replication initiation proteins [57]. Similarly, in viruses of the *Herpesviridae* family, including Epstein-Barr virus and human cytomegalovirus, replication origins are also defined by highly G-rich sequences with strong G4-forming potential [59]. These viruses maintain their double-stranded circular episomal DNA during latency by exploiting the host replication machinery. Given the extensive heterochromatinization of these viral genomes during latency [60], G4 formation at replication origins may be required to allow physical access of replication initiation factors, thereby ensuring viral genome maintenance.

Despite these advances, it remains unclear whether G4s can be considered determinants, at least for a subset of human replication origins and whether these origins share additional common features distinct from other G4-containing genomic loci. Also, exactly how G4s at origins would be stabilized in the context of double-stranded DNA remains elusive. Future studies will need to determine if G4s at replication origins, analogously to promoter G4s influencing chromatin looping in transcription, can regulate origin firing timing and nuclear organization, potentially through complex interactions with other epigenetic marks.

## When do G4s become pathological?

Alongside their regulatory functions, G4s can cause collateral damage and deeply affect genome stability if not efficiently processed. Although they are detected and processed throughout all phases of the cell cycle, G4s become particularly problematic during DNA replication. When stably folded, G4s can act as physical obstacles to replication fork progression that, if not properly resolved to unstructured single- or double-stranded DNA, can cause DNA breaks, as well as heritable epigenetic changes that can locally alter gene expression [61]. Consistent with this idea, G4-enriched promoters have emerged as mutational hotspots in genome-wide analyses [62]. Furthermore, stabilization of G4s using small-molecule ligands leads to increased replication stress and accumulation of DNA breaks in cells [63]. It is, however, important to highlight that not all G4-stabilizing ligands have the same effect on genome stability and that the mechanisms leading from G4 stabilization to DNA breakage are diverse and not fully understood. For example, while the high-affinity ligand PhenDC_3_ primarily leads to increased replication stress in cells, with limited DNA break formation [64], treatment with the G4-stabilizing ligand pyridostatin strongly induces cellular DNA damage that has been linked to transcription-dependent processes and is consistent with topoisomerase–DNA cleavage complexes at G4 sites [63]. Further support for the replication-associated toxicity of G4s comes from the existence of multiple helicases that are capable of unfolding these structures. Notably, several of these helicases, including FANCJ, PIF1, WRN, and BLM, are physically associated with the replisome, underscoring the need to resolve G4s during DNA synthesis. Accordingly, depletion of these helicases results in increased genome instability at G4 sites, an effect that is further exacerbated by G4-stabilizing ligands [11]. Together, these observations support the view that at least some G4s are not inherently deleterious, as they are normally unfolded at the replication fork through multiple, partially redundant pathways. Genome instability, therefore, appears to arise primarily when G4 processing is impaired, either through stabilization by ligands or through loss of factors involved in their resolution.

**Fig 4:**
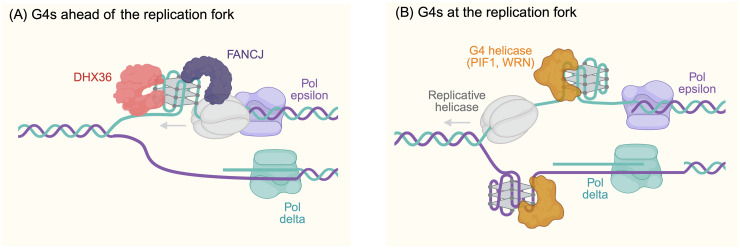
How do G-quadruplexes (G4s) hinder DNA replication? A. G4s encountered by the replisome ahead of the replicative helicase are primarily structures located at transcription start sites and telomeres. Pre-formed G4s are first encountered by the replicative helicase, which can stall if G4s are not properly resolved. The G4 helicases DHX36 and FANCJ can coordinately resolve this class of G4s. **B.** G4s formed at the sites of active replication on either the leading or the lagging strand are encountered by DNA polymerases epsilon and delta, and hinder DNA polymerization if not properly resolved. Replisome-associated helicases such as PIF1 and WRN have been implicated in the resolution of G4s generated during DNA replication. Created in BioRender. Gallina, I. (2026) https://BioRender.com/8kpz55s.

However, among the many G4s that have been detected across the genome, how can we determine which ones actively contribute to genome instability and which are benign or functional intermediates? During DNA replication, at least two distinct classes of G4s are likely to be encountered. The first class comprises promoter and telomeric G4s. These structures are already formed, often stabilized by R-loops, and they are encountered ahead of the replication fork (Fig 4A). These stable G4s have been recently suggested to be recognized and resolved in a replication-dependent manner by the helicases FANCJ and DHX36 and by a regulated pathway including the recombination proteins RAD51 and BRCA2 [65,66], although other helicases have also been implicated. Pre-formed G4s, particularly at promoters, are likely the most deleterious for genome stability as they may be hotspots for transcription–replication conflict and sources of DNA breaks when stabilized. A second class of G4s can form directly at the replication fork on stretches of single-stranded DNA generated on the parental DNA strand, particularly during mild replication stress (Fig 4B). These structures within the replisome seem to be recognized by a different set of helicases, including WRN and PIF1 [67], and may be sensed and processed as post-replicative damage, potentially exposing different therapeutic vulnerabilities. Further mechanistic studies will be necessary in this field as many questions remain to be answered. How do these two classes of G4s differ in how they impact genome stability? Does the interplay with R-loops render the structure more deleterious than G4s alone? What pathways process structurally different G4s and how is pathway choice regulated?

Exploiting the toxicity of G4 stabilization is emerging as a promising approach for cancer therapy, with G4 ligands entering early-phase clinical trials [68]. As cancer cells display consistently more G4s than healthy cells due to oncogene promoter amplifications and high transcription and replication stress [62], treatment with G4 ligands selectively sensitizes them, particularly when DNA repair is deficient, such as in BRCA2-negative breast and ovarian cancers. Importantly, similar to other chemotherapeutics, G4 ligands rely on induced replication stress to kill cancer cells, but, while conventional agents act diffusely across the genome, G4 ligands act at precise genomic sites, often enriched in regulatory regions. This offers a potential route to greater selectivity, especially in tumors that rely heavily on G4-resolving mechanisms, and may also expose vulnerabilities that traditional chemotherapies do not specifically exploit [69]. In light of this, identifying new pathways and proteins involved in G4 processing could support the development of combinatorial therapeutic strategies.

One further issue in targeting G4s with ligands is that the cell milieu is likely to influence the 3D conformation of G4s, which may not completely resemble that observed in vitro by X-ray crystallography and nuclear magnetic resonance, which are used to design G4-binding molecules. Therefore, physiologically relevant structural information will be crucial for the design of more potent and selective G4 ligands. In parallel, systematic studies on the off-target effects and toxicity of G4 ligands will need to be performed before clinical use of G4 ligands can become a valid option. Although G4 ligands such as QN-302 are in clinical trials as anti-cancer drugs [70,71], given that G4s are also abundantly detected in normal cells, it still remains an open question as to how G4 ligands will achieve sufficient specificity to be considered safe for clinical use. In this context, refinement of alternative G4-stabilizing approaches will be needed. Promising strategies for clinical translation could involve stabilizing the C-rich complementary strand of oncogenes using small RNA molecules or oligonucleotides, which stabilize G4s [72] and may help achieve promoter selectivity.

## Evolution of G4s and their functions

Computational predictions of G4s across a wide range of species suggest that these structures are not a recent evolutionary innovation. G4s are predicted in all kingdoms of life, indicating that, from an evolutionary perspective, they are ancient structural motifs that likely emerged early in the history of nucleic acids as a consequence of guanine chemistry. Over time, different organisms appear to have repeatedly co-opted G4s for regulatory functions, transforming them into functional elements [5].

In viruses, G4s are particularly intriguing. Despite their compact genomes and strong selective pressures, several families of DNA and RNA viruses maintain conserved G4-forming sequences in promoters, untranslated regions, and coding sequences [73], which are involved in regulating replication, transcription, translation, and genome packaging. Their conservation across viral strains hints at positive selection, despite stable secondary structures posing obstacles to polymerase activity. However, it remains an open question whether viral G4 folding is always dictated by viral fitness, or if it may be a viral response to host constraints. Retroviruses such as HIV illustrate the interplay between viral and host factors. HIV integrates into the host genome, where chromatin structure influences viral transcription. Multiple G4s form within the viral LTR promoter, fine-tuning or repressing transcription [74]. The virus also exploits host G4-binding proteins to optimize viral gene expression [75]. This highlights how the host environment can shape viral G4 evolution.

Interestingly, G4 distribution varies widely across organisms. Bacteria, for instance, have fewer and less stable G4s, which are often restricted to regulatory regions that control responses to environmental changes [76,77]. Their limited use may reflect differences in G/C content or the balance between the regulatory benefits and the risks of genome instability. Overall, G4s are an example of how a simple nucleic acid structure can evolve into a multifunctional regulatory element.

## Conclusions and future directions

Despite major advances over the past decade in understanding the physiological roles of G4s, substantial work remains to fully elucidate their biological functions and to harness their therapeutic potential, particularly in cancer therapy and antiviral strategies (see Box 1). A major challenge lies in the ability to safely and selectively target individual G4 structures, both to dissect their mechanisms of action and to develop effective therapeutic approaches.

**Box 1. Key outstanding questions in DNA G-quadruplex (G4) biology**

- **When and where do G4s actually form?**
- Which molecular and cellular features dictate whether predicted G4-forming sequences actually fold into G4 structures in cells at a given time? How are folding and unfolding dynamics coordinated to ensure functional relevance?
- **How do proteins recognize the right G4s?**
- Numerous proteins bind to or process G4s. What molecular features define their specificity, and how do cells coordinate the activities of these factors on G4 substrates?
- **Are G4s just transcriptional regulators or epigenetic features?**
- G4s are enriched in promoters and regulatory regions, but their causal role in controlling gene expression and whether they function as a bona fide epigenetic mark remains unresolved.
- **How much of a threat are G4s to DNA replication?**
- G4 structures can pose challenges to DNA replication. How frequently do they lead to replication stress or genome instability? And how have these effects shaped genome evolution?
- **Can G4s be therapeutically targeted safely and selectively?**
- Small molecules that stabilize G4s show promise, particularly in cancer models. Will it be possible to achieve the selectivity and safety needed for effective clinical use?

Because G4s are highly dynamic and influenced by numerous cellular factors, they may be more difficult to fine-tune than initially anticipated. This requires a deeper understanding of G4 conformations adopted in vivo and the exploitation of context-specific features to achieve selectivity. Upcoming strategies include directing ligands to specific G4s by coupling them to Cas9 variants [78], or by coupling G4 ligands to small RNAs or oligonucleotides that bind the strand complementary to the G4, thereby stabilizing specific structures [79]. In parallel, it will be important to develop more effective methods to destabilize G4s, which could be exploited to modulate gene expression. Genome-engineering technologies could also be employed to modify single G4s, or defined subsets of G4s, to obtain direct evidence of their roles in transcription, replication, and genome organization.

At the same time, single-cell, genome-wide approaches, such as single-cell CUT&Tag, are being used to investigate the coexistence of G4s with G4-binding proteins, R-loops, and other epigenetic marks [80]. These techniques will provide critical insights into their coordinated roles in genome regulation. Further development of such technologies will be essential for establishing causal relationships between G4s, chromatin architecture, and the establishment and inheritance of epigenetic marks during cell differentiation and development. Finally, studies of G4 evolution may shed light on how their functions emerged and may provide new opportunities to exploit these structures as targets for antiviral and antibacterial therapies.

In conclusion, G4 biology is a rapidly evolving field at the intersection between genome regulation, structural biology, and therapeutics. As experimental technologies continue to improve, a deeper understanding of G4 function and dynamics has the potential to open new possibilities for precision medicine and targeted regulation of gene expression.

## Abbreviations:

G4s: G-quadruplexes
TSSs: transcription start sites
